# Quality Management Interventions in Tracheotomy: A Retrospective Comparative Study

**DOI:** 10.7759/cureus.91415

**Published:** 2025-09-01

**Authors:** Julius Gerstmeyer, Aileen Spieckermann, Periklis Godolias, Christopher Ull, Christian Waydhas, Thomas A Schildhauer, Uwe Hamsen, Oliver Cruciger

**Affiliations:** 1 Department of Orthopedics and Trauma Surgery, Katholische Kliniken Bochum, Bochum, DEU; 2 Department of Orthopedics and Trauma Surgery, BG University Hospital Bergmannsheil, Bochum, DEU; 3 Department of Orthopedics and Trauma Surgery, St. Josef Hospital Essen-Werden, Essen, DEU; 4 Department of General and Trauma Surgery, BG University Hospital Bergmannsheil, Bochum, DEU; 5 Department of Orthopedics and Trauma Surgery, Universitätsmedizin Essen, Essen, DEU

**Keywords:** cannula, complication, complication management, quality management, safety, tracheotomy

## Abstract

Background

Tracheotomy is common in patients requiring long-term ventilation or neurological care. Managing these patients is challenging, as urgent cannula exchanges (CE) carry potentially life-threatening risks. To improve safety and standardize care, we implemented quality measures comprising a standardized bedside information sheet and mandatory staff training. This retrospective study evaluates their impact on complications in long-term tracheotomized patients.

Methods

A retrospective comparison study was conducted with the previous prospective cohort (pre) as a control and an intervention cohort (post) after implementing the stated quality measures. A chart review of adult tracheotomized patients treated between 2018 and 2020 in a 22-bed surgical ICU at a level 1 trauma center was performed. Each CE was documented with regard to its indications and complications, and descriptive and comparative analyses were performed.

Results

The study included 122 patients (pre: 49; post: 73). The post-group experienced fewer CEs per 100 days (2.65 vs. 4.58) and longer intervals between CEs (18 vs. seven days), with fewer planned exchanges. Although the post-group experienced higher tube occlusion and overall complications (pre 7.5% vs. post 8.5%), accidental decannulations were slightly lower. Respiratory complications were more common in the pre-group, while cardiocirculatory events predominated in the post-group.

Conclusion

Managing tracheotomized patients remains a significant clinical challenge. A standardized cannula information sheet and mandatory training may reduce the frequency of CEs and enhance patient safety.

## Introduction

Tracheotomy is a standard treatment for long-term ventilated or neurologically impaired patients. It is one of the most commonly performed procedures in ICUs [1]. Around 9% of all patients requiring mechanical ventilation receive a tracheostomy [2]. Early tracheostomy has been associated with benefits such as reduced ventilator-associated pneumonia (VAP) and earlier weaning in selected populations. Some studies, particularly in neurologically impaired or spinal cord-injured patients, have also reported increased ICU or long-term mortality associated with tracheostomy, potentially due to the underlying severity of illness [3,4]. Up to now, there are no standardized guidelines for the frequency of cannula exchanges (CEs). Urgent CEs are common and associated with potentially lethal complications. Especially, early tube exchanges are associated with increased rates of complications [5]. Management of several potential complications requires knowledge and expertise from everyone interacting with these patients. Most severely, accidental decannulation, tube occlusion, and the need for urgent CEs pose an immediate risk to patients’ lives and a challenge to healthcare providers [5-8].

Implementation of multidisciplinary teams and protocols has been shown to be beneficial by reducing the time to decannulation, length of stay, and rate of complications and promoting the use of speaking valves [9-12]. Our study group found a high rate of accidental decannulations and complications during urgent cannula changes in a prospective study on long-term tracheotomized patients [5]. Following this study, we implemented different quality management measures. Among others, a standardized information sheet at the bedside was developed and implemented. Positive results of standardized information usage in patient management and safety have been shown in many areas of practice [13,14]. Currently, it is unknown if the use of a standardized CE information sheet could lead to decreased rates of adverse effects by providing information in high-risk situations. The aim of this study was to evaluate the effect of structured quality management interventions on complication rates and the frequency of CEs in long-term tracheotomized patients. Specifically, we hypothesized that the interventions would reduce urgent CEs and patient-related complications by improving staff awareness and preparedness through standardized information sheets and mandatory training.

## Materials and methods

The study was conducted at the BG University Hospital Bergmannsheil Bochum, Department of General and Trauma Surgery, Bochum, Germany.

Prior to this study, our group conducted a prospective analysis of the incidences and complications of CEs in patients with spinal cord injury. The main findings included an overall complication rate of 12.3%, with patient-related complications of 8.5%, and 0.34 CEs per 100 days of observation. This cohort served as the intervention control group (pre) and was included in the tabulated comparison for a comprehensive comparison. Following that study, several steps to improve patient care were implemented. Using the Delphi method, a standardized information sheet was developed by four senior intensivists, containing information about the cannula in place, the date of the last change and tracheostomy, adverse events at previous changes, and the need for assistive tools when exchanging (Appendix 1). Mandatory training for healthcare providers on tracheal cannula management was organized and delivered by the study investigators. The training program consisted of theoretical teaching and practical discussions of CEs, including indications and complication management. Educational materials were made permanently available for review. The duration of the sessions varied depending on participant needs, and repeat sessions were available upon request to ensure continuous competency.

After implementing these quality management measures, a retrospective comparative analysis was conducted. A chart review of all patients treated between 2018 and 2020 on a surgical 22-bed ICU at a level 1 trauma center was performed (post). Patients either had a tracheostomy tube in place when admitted or were tracheotomized at the site.

The standardized information sheet was placed at the bedside. After each exchange, a new sheet was filled out. There was no protocol for the indication to exchange a cannula or technique, including the use of tools implemented. All CEs were performed by experienced physicians, defined as expert senior intensivists. For patients with recently (<21 days) established tracheostomies, exchanges were routinely carried out with the aid of an exchange device (e.g., airway exchanger) or, when judged safe, without such a device. Blinding of investigators during chart review was not applied, as this was neither feasible nor necessary within the context of a quality improvement project. The training curriculum is described above. CEs were documented as planned, urgent, or due to an emergency. A planned exchange was defined as an intervention with >48 hours of planning. An urgent exchange was defined as the need for a cannula change within the next few hours. Reasons for exchanges were defined as tube occlusion, decannulation, mechanical irritation of the patient, and weaning-associated (such as exchange for a placeholder or downsizing of cannula).

Indications such as patient comfort, patient safety, periodical change, or failure of the cannula, and parastomal complications such as bleeding or infection, were summarized under other reasons. Complications were subdivided into bleeding, respiratory, or circulatory. A respiratory adverse event was defined as desaturation (SpO2 <90%). Abnormal hemodynamics were divided into hyper/hypotension (>150; <90 mmHg) or brady/tachycardia (>120; <60 beats per minute) and the need for cardiopulmonary resuscitation (CPR).

This retrospective comparative study was performed with approval, and consent to participate was waived by the ethics commission (No. of Registration: 2024-388-f-S). The prospective observational study, examining the pre cohort, was also approved by the local ethics board (Approval 16-5889-BR).

Statistical analysis was performed using Excel (Microsoft® Excel® 2022, Version 16.64, Microsoft Corporation, Redmond, WA, USA). Descriptive analyses are presented as mean, standard deviation (SD), and median where appropriate. The number of changes per 100 days of treatment was calculated by the incidence and total number of treatment days. For independent variable analysis, a chi-square test was used, and for continuous variables, a T-test was used. To compare proportions, a Z-test was used. For numbers below 10, no statistical analysis was performed. No formal sample size calculation was performed. All eligible patients treated during the defined study period (2018-2020) were included to maximize case identification and ensure comprehensive representation of clinical practice during the intervention phase.

## Results

This retrospective comparison study included a total of 122 patients, 49 patients in the pre and 73 in the post-cohort (Table 1). One patient was excluded from the post-cohort due to an incomplete dataset. There was no statistical difference in both age and sex distribution. The median age of patients in the pre-group was 64 years (IQR: 52-72), with 61 years (IQR: 52-71) in the post-group. A spinal cord injury was present in 75% of the post-group compared to all patients in the pre-group. There was no significant difference between the groups for both mean observation period per patient (post 67 days (34-100); pre 50 days (22-101)) and total days of observation (post 5358 days; pre 3191 days).

**Table 1:** Patient demographics Values are presented as median and interquartile range or number (%). The p-values indicate statistical significance of differences between the two groups. Continuous variables were compared using independent t-tests, and categorical variables were compared using chi-square tests.

Demographics	Pre (n=49)	Post (n=73)	p-value
Male	40 (82)	59 (81)	0.288
Age (years)	64 (52-72)	61 (52-71)	0.257
Patient with spinal cord injury	49 (100)	55 (75)	
Total days of observation	3191	5358	0.177
Duration of observation per patient	50 (22-101)	67 (34-100)	0.180

Table 2 summarizes the data on cannula changes. In total, 142 CE were documented in the post-group and 146 in the pre-group. In the post-group, 83.8% of CEs were performed on patients with a spinal cord injury (SCI), whereas all CEs were performed on SCI patients in the pre-group. There were several statistically significant differences between the study groups (p<0.001). In the post-group, the median interval between CE was longer (18 days (IQR 6-70) vs. seven days (IQR 2-15)), with fewer planned CE (41.5% vs. 54%) and fewer CE per 100 days of treatment (2.65 vs. 4.58). These findings resulted in significantly fewer planned and urgent CEs per 100 days of treatment (1.1 vs. 2.48; 1.01 vs. 2.10), although urgent CEs were more frequent in the post-group (58% vs. 46%). The total number of CEs per 100 treatment days was 4.58 (95% CI: 3.87-5.39) in the pre-group and 2.65 (95% CI: 2.23-3.12) in the post-group.

**Table 2:** Cannula changes Values are presented as median and interquartile range or number (%). The p-values indicate statistical significance of differences between the two groups. Continuous variables were compared using independent t-tests, and categorical variables were compared using chi-square tests.

Cannula changes	Pre (n=146)	Post (n=142)	p-value
Cannula exchanges in patients with spinal cord injury	146 (100)	119 (83.8)	
Days between cannula exchanges	7 (2-15)	18 (6-70)	<0.001
Planned cannula exchanges	79 (54)	59 (41.5)	<0.001
Urgent cannula exchanges	67 (46)	83 (58)	<0.001
Exchanges of cannula per 100 days of treatment	4.58	2.65	<0.001
Exchanges in patients with spinal cord injury per 100 days of treatment	4.58	2.22	<0.001
Planned exchange of cannula per 100 days of treatment	2.48	1.1	<0.001
Urgent exchange of cannula per 100 days of treatment	2.10	1.01	<0.001

Indications for CE, summarized in Table 3, showed several significant differences between the two groups. Tube occlusion was more frequent in the post-group (25.4% vs. 15.1%), whereas accidental decannulation was more common in the pre-group (21.2% vs. 16.9). CE were performed due to mechanical irritation and weaning associated in 10.6% and 14% respectively. However, these parameters were not specified in the pre-study. Overall, other reasons were significantly more frequent in the post-group (23% vs. 8.2%).

**Table 3:** Indications for cannula change Values are presented as median and interquartile range or number (%). The P-values indicate statistical significance of differences between the two groups. Continuous variables were compared using independent t-tests, and categorical variables were compared using chi-square tests. *Variable was not included in the pre-study.

Indications for cannula change	Pre (n=146)	Post (n=142)	p-value
Tube occlusion	22 (15.1)	36 (25.4)	<0.001
Mechanical irritation	*	15 (10.6)	
Weaning associated	*	20 (14)	
Accidental decannulation	31 (21.2)	24 (16.9)	<0.001
Other	12 (8.2)	33 (23)	<0.001

In Table 4, complications were subcategorized and described. We found a complication rate of 8.5% in the post-group, which is significantly higher compared to the pre-group (7.5%). CE with respiratory complications were statistically more common in the pre-group (76.9% vs. 58.3%), while cardiocirculatory complications were more frequent in the post-group (66.7% vs. 23.1%). Notably, CPR was required in two patients in the post-group and one patient in the pre-group. Although the post-group had fewer reported patient-related complications per 100 days of treatment (0.22 vs. 0.34), the difference between the study groups was not statistically significant.

**Table 4:** Complications Each cannula exchange may have more than one complication; therefore, category percentages do not add up to 100%. Values are presented as median and interquartile range or number (%). The p-values indicate statistical significance of differences between the two groups. Continuous variables were compared using independent t-tests, and categorical variables were compared using chi-square tests. *Variable was not included in the pre-study.

Complications	Pre (n=146)	Post (n=142)	p-value
Exchanges with patient-related complications	11 (7.5)	12 (8.5)	<0.001
Exchanges with respiratory complications	10 (76.9)	7 (58.3)	<0.001
Exchanges with cardiocirculatory complications	3 (23.1)	8 (66.7)	<0.001
Tachycardia	1	1	
Bradycardia	2	2	
Hypertension	*	2	
Hypotension	1	5	
Cardiopulmonary resuscitation (CPR)	1	2	
Exchanges with patient-related complications per 100 observation days	0.34	0.22	0.297
Respiratory complications per 100 observation days	0.31	0.13	0.067
Cardiocirculatory complications per 100 observation days	0.09	0.14	0.490

## Discussion

The aim of our study was to evaluate the impact of a standardized CE information sheet on the rate of complications experienced by long-term tracheotomized patients. Literature emphasizes the challenges and risks associated with a tracheotomy, particularly the management of cannula-related complications. Recent publications showed the benefits of standardized care pathways in the reduction of length of stay, life-threatening respiratory events, and patient outcomes [11,12]. Reports on rates of CEs are particularly rare. Up to now, there is no international guideline on routine CEs. Currently, CEs are based on individual patient conditions, clinical settings, and professional judgment.

Published data of our pre-group showed a rate of CE of 4.58/100 days of observation [5]. The current study found a significantly lower rate at 2.65/100 days of observation (p<0.001), with a similar finding if accounting for only urgent exchanges (pre 2.1 to post 1.01). Although lacking a statistically significant difference, the post-group showed a longer observation period (5358 days to 3191 days) but almost the same number of CE (142 to 146). This resulted in a longer median days between CE (18 (6-70) days compared to a median of 7 (2-15) days in the pre-group). The results of the present study show a greater IQR, indicating that some patients required exchanges more frequently and at different time points than others. A similar detail was observed in the pre-study, with urgent CE being more frequent in the first eight weeks after tracheostomy. Previous studies also indicated a high incidence of CE within the first week after tracheostomy due to a variety of reasons [15,16]. Another factor influencing the rate of CE is differences in study populations, as the pre-study only included SCI patients. Aside from SCI, especially cervical spine injuries, there are several indications for a tracheostomy. Indications include airway obstruction, laryngeal paralysis, neoplasia, altered mental status, and long-term ventilation [2,17]. These patients may have a different risk profile for CE, as our results show a lower rate of CE per 100 days of treatment at 2.22 compared to 2.65 in the general population. However, a reduction in the number of CE may reduce the number of exchange-related complications.

The authors assume that the observed lower rates in CE are mainly due to the implemented mandatory training for healthcare providers and the information sheet at the bedside. This resulted in a potential increase in experience in cannula management and awareness of potential complications associated with CE. This may have led to a more critical indication of CE and thus overall less CE. This hypothesis is supported by significantly fewer planned CE performed in the post-group (41.5% vs. 54%). Additionally, we found significantly more urgent CE in the post-group. This finding might be elucidated by a shift towards urgent CE as less planned CE was performed. Critical indications for CE might have led to cannulas being longer in place (median days between CE: 18 (6-70) days compared to a median of 7 (2-15) days in the pre-group), but still requiring a change later in time. Thus, the proportion of urgent CE may have increased. 

Furthermore, tube occlusion as an indication for CE was more frequent in the post-group (25.4% vs. 15.1%; p=<0.001), whereas accidental decannulation was more common in the pre-group (21.2% vs. 16.9%; p=<0.001). Cannulas being longer in place in the post-group may account for the increase in tube occlusion observed due to the increased likelihood of secretions or debris accumulation. However, the reduction of accidental decannulation might be an effect of improved management. This finding is in accordance with previously published studies. Following implementation of standardized trach trail education, Cherney et al. found, among other complications, a reduction in accidental decannulations [11]. Improved cannula management may not affect each indication for CE in the same way, but result in an overall decreased number of CE. As advised by Garuti et al., our findings also emphasize the need for multidisciplinary specialized caretakers when treating tracheotomized patients, especially those with SCI [18].

In the post-group, we observed a significantly higher complication rate of 8.5% compared to 7.5% in the pre-group. Despite the absolute increases, the rate of complications per 100 days was lower in the post-group (0.22 vs. 0.34), suggesting that our interventions still confer a net benefit by reducing life-threatening events. However, it is noted that the rates did not differ significantly between the groups (p=0.297), with statistical power limiting this finding due to the relatively small number of events. Comparing the subgroup analysis of disorders, the numbers for circulatory disorders quadrupled while respiratory disorders declined in the post-group. This fact might be attributed to effects resulting from mandatory training of healthcare providers. Previously, Masood et al. also showed a reduction in life-threatening respiratory events following implementation of a tracheostomy care protocol [12]. Furthermore, the increased rate of cardiocirculatory complications in the post-group may be elucidated by several factors. Firstly, it is noted that in the pre-group, hypertension was not included as a complication. This may lead to an overrepresentation of cardiovascular complications in the post-group. Secondly, more urgent exchanges were performed in the post-cohort. These may have been performed later with patients being in worse condition, especially in complex emergency situations like tube occlusion. This may result in hypoxemia and cardiovascular complications. Respiratory complications might have been detected earlier, thus preventing cardiocirculatory disorders. Improved complication management and increased utilization of tools such as bronchoscopy or the use of smaller cannulas as a means of last resort may elucidate these findings. Notably, cardiocirculatory disorders led to the need for CPR in both studies. This emphasizes the potential life-threatening aspect of complications during cannula changes.

Several limitations must be acknowledged. First, the retrospective pre-post design carries an inherent risk of confounding, as unmeasured factors may have contributed to the observed outcomes. Second, variability in the intensity and duration of the training intervention may have influenced the results. Third, the relatively short post-intervention observation period does not allow for conclusions regarding the long-term sustainability of the effects. Furthermore, the reliance on chart reviews, which may have incomplete or inconsistent documentation, may also introduce bias.

In addition, several further limitations must be considered. The limited number of complications restricts statistical power and may have led to underestimation of true effect sizes. Furthermore, unmeasured confounding factors, including staff experience, could have influenced the results. While we provided detailed descriptions of the intervention, reproducibility may still be limited as the intervention was embedded in a specific institutional setting. Taken together, these limitations warrant cautious interpretation of our findings and underline the need for prospective studies with larger samples.

Although indications for tracheotomies are heterogeneous, variability between these groups may also influence the overall comparability of the results. The effectiveness and adherence to the mandatory training provided to healthcare providers could vary and may affect the results. These factors should be addressed in future prospective studies with longer follow-up.

## Conclusions

Management of tracheotomized patients remains a challenge. Our findings suggest that standardized information sheets and mandatory training may reduce the number of urgent CEs and accidental decannulations. However, the overall complication rate was not reduced, and due to the retrospective pre-post design, causal inferences are limited. The implemented quality measures may have increased the competence and confidence of healthcare providers in managing tracheostomized patients, leading to more informed and cautious decision-making. These results should therefore be interpreted cautiously and require validation in prospective, controlled studies before definitive conclusions can be drawn regarding the impact on patient safety.

## Figures and Tables

**Figure 1:**
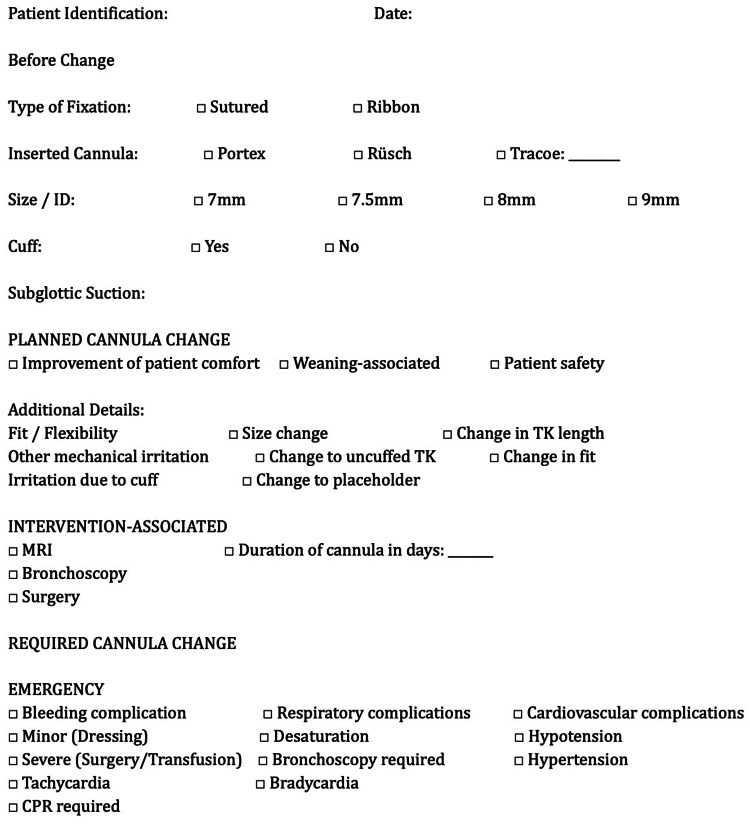
Standardized information sheet (part 1)

**Figure 2:**
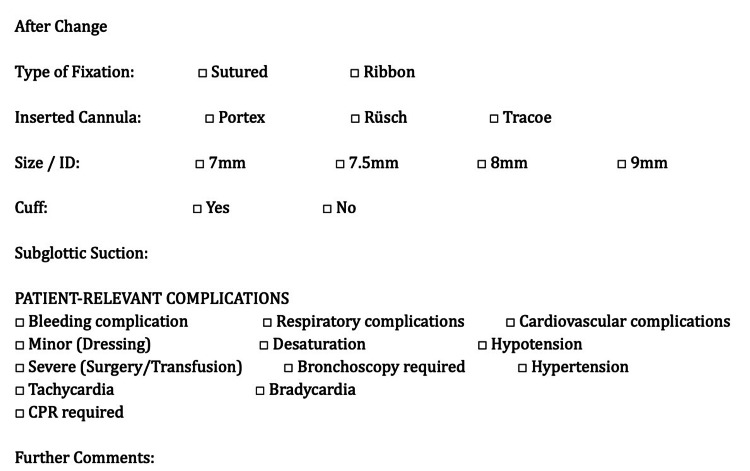
Standardized information sheet (part 2)

## Appendices

Appendix 1
